# Protocol for a systematic review: understanding the motivations and barriers to uptake and use of female-initiated, primary biomedical HIV prevention technologies in sub-Saharan Africa

**DOI:** 10.1186/s13643-015-0096-1

**Published:** 2015-08-19

**Authors:** Robyn Eakle, Caitlin Jarrett, Adam Bourne, Jonathan Stadler, Heidi Larson

**Affiliations:** Wits Reproductive Health and HIV Institute, Johannesburg, South Africa; London School of Hygiene and Tropical Medicine, London, UK

## Abstract

**Background:**

Women in sub-Saharan Africa are disproportionately affected by high rates of HIV, yet relatively few products exist for female-initiated HIV prevention. New antiretroviral (ARV)-based prevention options could present opportunities for women to expand their HIV prevention choices; however, acceptability and adherence play a key role in the effectiveness of these products and implementation is still in early stages. To better understand which HIV prevention options might best serve women in sub-Saharan Africa, how and why, this review will explore qualitative evidence from clinical trials and implementation studies alike using a meta-ethnographic approach to synthesise data and interpret results.

**Methods/design:**

This systematic review will use a meta-ethnographic approach to analyse qualitative data extracted from multiple studies featuring actual use of female-initiated technologies for HIV prevention. The search strategy will be applied in seven databases and papers will be selected using strict inclusion and exclusion criteria. The review will closely follow the guidance set forth by preferred reporting items for systematic reviews and meta-analyses (PRISMA) and Centre for Reviews Dissemination (CRD) where the guidance applies to qualitative data. Two reviewers will review all papers during the paper selection phase, with consultation from a third reviewer to confirm consensus. All papers included in the review will be read and analysed by two reviewers. The final analysis will be conducted by three primary reviewers with additional input from all other authors.

**Discussion:**

With new HIV prevention technologies currently in early implementation phases and still more on the horizon, there is much to learn about how best these products may be delivered. A review such as this could help to inform the real-world implementation of the next wave of new HIV prevention technologies such as ARV-based oral pre-exposure prophylaxis (PrEP).

## Background

New HIV infections in sub-Saharan Africa persist at high rates where women, in particular, are disproportionately affected [1]. In South Africa, a survey published by the Human Sciences Research Council in 2012 estimated the national prevalence rates among women between the ages of 20 and 49 to be between 17.4 and 31.6 % [2]. Current methods for HIV prevention have taken the field only so far, but to further reduce new infections, new options for prevention are needed.

There are several approaches men and women can take to prevent the acquisition or transmission of HIV. Currently, these include the following: the use of male or female condoms; medical male circumcision; or the use of post-exposure prophylaxis (PEP), an antiretroviral (ARV) drug-based regimen given after suspected exposure to HIV. The majority of these prevention options, aside from PEP, are either entirely or partially controlled by men, or their use is dependent upon male acceptance. Male condoms require mutual agreement for use, and while the female condom can be initiated by women, it difficult for them to do so covertly. Additionally, research has suggested that female condoms are often difficult to access, and not always acceptable or easy to use [3]. PEP has typically only been available or accessible for specific circumstances, such as post-rape care [4], and the limited implementation suggests there may be significant intervention capacity issues relating to the training and education of healthcare providers [4, 5]. The diaphragm has been tested as an option for preventing HIV infection, and shown to have no effect [6], although efforts have been renewed to revive it as an option by redesigning the product and adding a microbicide. Studies published in the last 5 years [7, 8] indicate that ARV-based HIV treatment has also been shown to have a powerful secondary prevention effect by the suppression of an infected individual’s viral load; however, the population-level impact of this will take time and is unlikely to control the epidemic on its own [9].

A new primary prevention option utilising ARV-based medication has shown significant promise. Development of a variety of products (including pills, intravaginal microbicide gels, films and intravaginal rings) has yielded a new first-generation option in the form of a daily pill-based regimen, called pre-exposure prophylaxis (PrEP). Six recent clinical trials testing oral PrEP have shown varying levels of overall efficacy [10–13], from 44 to 75 %. At present, however, the indication is currently only registered in the USA. The varying degrees of efficacy from the oral PrEP trials can mostly be attributed to adherence, or consistent and correct use of the products [14]. The FEMPrEP trial, consisting only of women, was stopped for futility, while the VOICE trial was partially stopped for futility, with the final arm continuing to a flat result [15, 16]. These findings have caused researchers and implementers alike to question whether adherence-dependent ARV-based prevention options would actually be effectively taken up by women and make a significant contribution to HIV prevention. Analyses of qualitative and mixed method research from these trials are underway, along with additional follow-up studies to better understand why women did not use the products. Two papers already published on this area have revealed important insights as to the reasons women did not use the products, including the following: misaligned incentives for participating in the trials (money and access to better services); ambivalence about the research resulting in non-use of the products; a lack of understanding of the need to accurately report actual product use; and misperceptions of personal risk of HIV [17, 18]. This has shown that non-use of the products often had little to do with the products themselves and more to do with contextual issues, which may mean that oral PrEP could still be a viable prevention option if offered to suitably motivated users. This perspective is reinforced by recent results from the FACTS001 trial, which evaluated the efficacy of tenofovir-based intravaginal microbicide gel and established the gel not to be efficacious in preventing HIV acquisition, largely as a result of complex behavioural/social factors, as well as possible biological ones [19].

Given the complexities highlighted in the PrEP efficacy trials about delivering and implementing new prevention options, it is important to understand women’s perspectives and what factors determine the use of emerging biomedical technologies. This is essential not only to understand how existing HIV prevention options may be better implemented, but also to inform the delivery of the next wave of technologies, such as oral PrEP, as well as other new products further upstream in the development pipeline, such as vaginal rings containing microbicides and injectables. To date, a number of studies have explored the acceptability of HIV prevention technologies, including those already licenced for widespread use and those in development or clinical trial phases, as well as other contextual factors that function as motivators or barriers to their use. Studies producing qualitative data, like those stemming from the failed oral PrEP trials, are imperative for understanding what people think, as well as how and why they make decisions regarding uptake of HIV prevention options.

This systematic literature review will explore, synthesise, interpret and present the key factors that motivate or deter uptake and use of prevention technologies for women in sub-Saharan Africa. Such a review can help to inform the development and rollout of new technologies as they become available. This review will focus on female-initiated technologies, which are those women administer themselves, and can be technologies already available in the market or in the development phase. Data included in the review will be drawn from studies of actual product use, rather than hypothetical investigations. Technologies included in the review will include the following: PEP, PrEP, vaginal microbicides, the diaphragm and the female condom. While the diaphragm is not currently an accepted, efficacious method for preventing HIV, the research conducted around experiences with use will be included in this review as these may provide transferable insights into other product use. Qualitative research is best suited to the exploration of personal experience and decision making. Given its focus on the motivations and barriers to the uptake and use of technologies, the review will focus on the synthesis and analysis of qualitative data only, utilising a meta-ethnographic approach.

## Methods/design

This is a systematic review using a meta-ethnographic approach to analysis. As such, the review will include only qualitative data extracted from studies identified through intensive, systematic searches, and will closely follow the guidance set forth by the preferred reporting items for systematic reviews and meta-analyses (PRISMA) statement and the Centre for Reviews and Dissemination (CRD). This review has not been registered on the international database of prospectively registered systematic reviews in health and social care (PROSPERO) as since it is a review of qualitative evidence, it does not qualify. As a result, there is no PROSPERO number associated with this review.

The primary objective of this review is to identify and understand the motivations and barriers affecting uptake and use of female-initiated, primary biomedical HIV prevention technologies for women in sub-Saharan Africa. The review will incorporate the following secondary objectives:

- To identify types of studies from which data are extracted (e.g. stand-alone qualitative/social science, implementation or service delivery evaluation, or clinical trials)
- To describe elements of uptake and use of specified technologies by sub-population (e.g. female sex workers, victims of rape, or young women)
- To describe factors that influence uptake and use by intervention
- To identify priorities for future research

### Search strategy

The search strategy incorporates four primary concepts: HIV; uptake and use; qualitative research; and sub-Saharan Africa. Search strategy concepts have been constructed using an iterative process. Search terms will be adapted across databases to account for variations in subject headings. The following databases will be included in the search: Africa-Wide Info, CINAHL, Embase, Global Health, MEDLINE, PsycINFO, and Web of Science. The MEDLINE search strategy was developed as the primary search strategy template and will be adapted for the other databases.

### Inclusion and exclusion criteria

Papers will be selected during the screening process using the following inclusion criteria:

- Population: adult women, age 18 and above
- Intervention: female-controlled, biomedical—PrEP/microbicides, PEP, female condom, and diaphragms
- Comparator: not relevant
- Outcome: narrative on the motivations or barriers to HIV prevention uptake and use
- Study design: qualitative studies—interviews or focus groups
- Study type: studies must be based on primary research where products have been made available for actual use
- Location: sub-Saharan Africa

Exclusion criteria are as follows:

- Any papers published on research occurring before 2003
- Reviews
- Any studies based on hypothetical or potential use of products not made immediately available at the time of the research
- Any data not from the women’s point of view as the user
- Any research from the perspective of HIV-positive women
- Any research based on secondary prevention technologies or interventions (eg. PMTCT)

Papers will be excluded from the review if they do not meet all of the criteria above. Papers will not be excluded according to a quality assessment, per se, but will be evaluated according to principles and practices as outlined below. There will be no limitation by language, and grey literature will be included if identified through the database searches or through consultation with relevant experts.

The concept of experience of actual use is central to this review. As such, we will include data from studies conducted in both trial and implementation settings. It may be argued that incentives could be quite different in a trial setting where participants are paid to come to a clinic as compared to a ‘real-world’ setting where patients come when they feel they need to. From this perspective, trial participants could be more incentivised to attend the clinic. However, in the case where new prevention options proven to protect against HIV are available, the accessibility of the product or intervention may be the incentive to come to the clinic. Clearly, there are a variety of motivators for engaging in health-related behaviour, which we hope to explore in the context of female-initiated HIV prevention. In this review, we hypothesise that some of the experiences of actually *using* the products should be similar regardless of initial motivation. Additionally, some HIV prevention trials were stopped early or ended with a flat result because of non-use. In this paper, we aim to elucidate the issues leading to non-use across both the trial and the clinic settings, and hypothesise that reasons for non-use are likely to be similar. We will also compare between the two setting types to highlight any differences.

The year 2003 was chosen as the cutoff point for selecting papers as it marks the establishment of the president’s emergency plan for AIDS relief (PEPFAR) and the wide-scale introduction of antiretroviral therapy (ART) for HIV treatment in Africa. With the introduction of effective medication regimens, general population views of HIV and the means by which its transmission could be managed began to change [20]. The rollout of ART in Africa significantly changed the approach to the epidemic in sub-Saharan Africa which, until this time, largely consisted of efforts to promote condom use and HIV testing with the aim of preventing new infections; this had limited success as people testing HIV-positive could not access life-saving medication. Although treatment was not uniformly rolled out simultaneously in all countries in 2003, the launch of PEPFAR and increased access to ART renewed hope and motivation to prevention efforts and is attributed to the turn of the epidemic in Africa [21].

### Screening process

All references will be uploaded into a reference manager database and duplicates removed before starting the paper selection process. After de-duplication, all papers will be reviewed by title and abstract according to the inclusion and exclusion criteria by two reviewers. Any discrepancies between reviewers will be discussed and mediated by a third researcher. Once papers have been selected by title and abstract, three reviewers will review papers by full-text according to the inclusion criteria. Decisions regarding inclusion of papers from the same study will occur through discussion with all three reviewers on a case by case basis. All papers selected by the reviewers will be discussed by all three reviewers to reach consensus and confirm inclusion.

### Data extraction, analysis and synthesis

This review will be conducted as a meta-ethnography. This process of analysis will follow the principles set out in Noblit and Hare [22], but will use a form of this method as adapted more recently by several researchers [23–25]. Specifically, this adapted form of meta-ethnographic synthesis allows for qualitative data collected through methods beyond ethnography, such as interviews and focus groups, to be combined and interpreted to derive meaning and understanding of data across different types of studies.

Once papers for inclusion have been finalised after full-text review, reviewers will be assigned papers for data extraction according to the data extraction tool developed for the review. A thematic framework will also be constructed through the data extraction phase in an iterative fashion, with input from all reviewers. A minimum of two reviewers will extract data from each paper; consensus will be reached with input from the third reviewer on any differences in extracted data. The thematic framework will be used to build the meta-ethnographic constructs which comprise three layers: the perspective of the participants, the perspective of the authors, and the perspectives of the researchers conducting this review. These layers are often fluid and may overlap where the perspectives of the authors cannot always be divided from those of the participants, but eventually, these two initial layers lead into the construction of the final layer in which new understanding can be developed by looking across all of the data at once.

### Quality assessment

In contrast to quantitative research, there has been considerable debate over how to assess the quality of qualitative research [26–29]. Inherent to qualitative research is a flexible, iterative, and pluralistic process which is guided by the context in which it is conducted and by the research subjects themselves. Several authors have argued that it is not typically an undertaking easily standardised by checklists, which could actually hinder the creative nature of the work. Instead, there are accepted practices, or fundamental principles, by which one can analyse the research [27, 30]. An approach often adopted is the ‘the weight of evidence review’, which first establishes elements of good research, and those elements are then taken into account when looking across all of the papers included in a review [31]. This enables an assessment of the quality of the literature, so that the review can be judged as a whole rather than evaluating individual elements of each paper. In this review, a combination of the fundamental principles and weight of evidence review may be employed to judge the overall level of quality of papers included.

### Anticipating limitations and bias

As with any research study, the authors recognise that there will be limitations to this particular review.

Some components of the qualitative research included in individual studies may have been excluded from publication due to limits enforced by the publisher. Often, in-depth descriptions of methods, and development of the research and process through which it was undertaken, are left out of the publications, making it difficult to assess quality of the research process and the awareness of the researchers’ positions within their own research (reflexivity). Inevitably, these exclusions would affect the potential scope of qualitative evidence available for this review.

It is possible that some research may be absent altogether from the searchable and published literature. Research has shown that around 44 % of conference abstracts on qualitative research are translated into papers published in journals—which is about the same rate as for quantitative research [32]. Additionally, while this review will not exclude grey literature, the constraints of web-based searching and the lack of systematic, online grey literature indexing means it is difficult to determine whether our search has successfully captured all relevant grey literature.

## Discussion

This systematic review, using a meta-ethnographic approach for analysis, is the first of its kind to synthesise qualitative data across a wide and complex body of literature in this subject area. The ultimate aim of this study is to learn from research already conducted on women’s experiences of HIV prevention technologies and their motivations to take up and use them (and barriers to doing so), in order to inform the real-world implementation of the next wave of new technologies such as oral PrEP. Ultimately, these findings can help with future programming design, decisions and research to optimise delivery of existing and new HIV prevention interventions for women across sub-Saharan Africa.

## Abbreviations

ART: antiretroviral therapy
ARV: antiretroviral
CRD: Centre for Reviews Dissemination
FEMPrEP: pre-exposure prophylaxis trial for HIV prevention among African women
HIV: human immunodeficiency virus
PEP: post-exposure prophylaxis
PMTCT: prevention of mother to child transmission
PrEP: pre-exposure prophylaxis
PRISMA: preferred reporting items for systematic reviews and meta-analyses
PROSPERO: international database of prospectively registered systematic reviews in health and social care
VOICE: vaginal and oral interventions to control the epidemic
